# Expression- and splicing-based multi-tissue transcriptome-wide association studies identified multiple genes for breast cancer by estrogen-receptor status

**DOI:** 10.1186/s13058-024-01809-6

**Published:** 2024-03-21

**Authors:** Julian C. McClellan, James L. Li, Guimin Gao, Dezheng Huo

**Affiliations:** 1 Department of Public Health Sciences, University of Chicago, Chicago, IL 60637 USA; 2 Section of Hematology & Oncology, Department of Medicine, University of Chicago, Chicago, IL 60637 USA

**Keywords:** Breast, Cancer, TWAS, Estrogen, Receptor, Splicing

## Abstract

**Background:**

Although several transcriptome-wide association studies (TWASs) have been performed to identify genes associated with overall breast cancer (BC) risk, only a few TWAS have explored the differences in estrogen receptor-positive (ER+) and estrogen receptor-negative (ER-) breast cancer. Additionally, these studies were based on gene expression prediction models trained primarily in breast tissue, and they did not account for alternative splicing of genes.

**Methods:**

In this study, we utilized two approaches to perform multi-tissue TWASs of breast cancer by ER subtype: (1) an expression-based TWAS that combined TWAS signals for each gene across multiple tissues and (2) a splicing-based TWAS that combined TWAS signals of all excised introns for each gene across tissues. To perform this TWAS, we utilized summary statistics for ER + BC from the Breast Cancer Association Consortium (BCAC) and for ER- BC from a meta-analysis of BCAC and the Consortium of Investigators of Modifiers of BRCA1 and BRCA2 (CIMBA).

**Results:**

In total, we identified 230 genes in 86 loci that were associated with ER + BC and 66 genes in 29 loci that were associated with ER- BC at a Bonferroni threshold of significance. Of these genes, 2 genes associated with ER + BC at the 1q21.1 locus were located at least 1 Mb from published GWAS hits. For several well-studied tumor suppressor genes such as *TP53* and *CHEK2* which have historically been thought to impact BC risk through rare, penetrant mutations, we discovered that common variants, which modulate gene expression, may additionally contribute to ER + or ER- etiology.

**Conclusions:**

Our study comprehensively examined how differences in common variation contribute to molecular differences between ER + and ER- BC and introduces a novel, splicing-based framework that can be used in future TWAS studies.

**Supplementary Information:**

The online version contains supplementary material available at 10.1186/s13058-024-01809-6.

## Background

Estrogen receptor-positive (ER+) and estrogen receptor-negative (ER-) breast cancer (BC) not only have markedly different clinical prognoses and treatment decisions [1–3], but also are molecularly and etiologically distinct. Several non-genetic risk factors that differ between ER + and ER- BC including age at first birth, nulliparity, obesity in younger women, and age of menarche have been identified in previous studies [4, 5]. Genetic differences of breast cancer by ER status were first characterized between *BRCA1* and *BRCA2*-related tumors [6, 7], with *BRCA1*-related tumors predominantly lacking ER expression [8–10]. While rare mutations in some breast cancer susceptibility genes such as *BRCA1* and *BRCA2* with moderate to high penetrance have been shown to play a distinct role in breast cancer etiology by ER status [11–13], genome-wide association studies (GWAS) have additionally reported that common variants associated with breast cancer risk differ between ER subtypes [14–21]. Though these GWAS have indicated common variants may contribute to genetic differences between ER + and ER- BC, the genes by which these variants act through to impact the risk of developing each ER subtype have not been fully explored.

Transcriptome-wide association studies (TWAS) have recently emerged as an approach to explore how predicted expression of genes by common variants associates with various diseases by incorporating both summary statistics from GWAS and expression prediction models using information of expression quantitative trait loci (eQTLs) [22–24]. By training predictive models of gene expression on a reference panel of individuals for whom gene expression and single nucleotide polymorphism (SNP) data are available, TWAS studies bypass the need to obtain gene expression data for larger cohorts where only SNP data is available. As a result, TWAS studies have been shown to comparatively have more power than GWAS studies in identifying genes that potentially impact complex traits [25]. Most previous TWAS performed to study breast cancer have focused on overall breast cancer risk [26–29] with only a few studies that have explicitly explored ER- BC or stratify their analysis by ER status. These past studies that explore how gene associations differ by ER status were in European ancestry breast cancer TWAS [30, 31], or meta-analysis of multiple ancestry TWAS of breast cancer [32, 33]. In total, these studies have identified 22 genes that associate with ER- BC and 69 genes for ER + BC (Table S1 in Additional file 1).

Though the aforementioned studies established an initial framework for performing TWAS by ER status, there are several shortcomings. First, all these studies utilize expression prediction models to perform TWAS analyses, which do not explicitly account for alternative splicing events within each gene. Splicing is markedly important to include in subtype-specific TWAS analyses since dysregulation of alternatively spliced transcripts has been shown to play a considerable role in BC etiology in prior studies [34–36]. Second, these previous TWAS studies have been primarily focused on studying the association between BC risk and gene expression in breast and blood tissues. The effects of gene expression in tissues other than breast tissue on breast cancer risk have thus far not been addressed for either ER + or ER- tumors. Third, the expression prediction models used in these prior studies have been based on modest sample sizes, which limits their power to detect associations. In this study, we work to address these limitations by performing a comprehensive subtype-specific TWASs of ER- and ER + breast cancer that utilize information of splicing quantitative trait loci (sQTLs) in addition to eQTL information across multiple tissues from the latest version of GTEx models (v8) to increase our power to detect breast cancer susceptibility genes by ER status.

## Methods

**Subtype-specific GWAS summary statistics for women with European ancestry.** We obtained ER + BC summary statistics that have been previously generated from 69,501 ER + BC cases and 105,974 controls who are women with European ancestry in the Breast Cancer Association Consortium (BCAC) [37, 38]. As described in previous studies, these summary statistics for ER + BC were obtained by using an inverse-variance fixed-effects meta-analysis of BCAC participants who were genotyped using the OncoArray, participants who were part of the Collaborative Oncological Gene-Environment Study (iCOGS), and participants from 11 other BC GWAS studies within BCAC. We obtained ER- summary statistics for a total of 30,882 ER- cases and 115,468 controls who are women with European ancestry by performing a meta-analysis of 21,468 ER- cases and 105,974 controls from BCAC and 9,414 BC cases and 9,494 controls from the Consortium of Investigators of Modifiers of BRCA1 and BRCA2 (CIMBA) [39] using an inverse variance-based approach with the METAL software package [40].

**Expression and splicing prediction models.** Both overall gene expression and intron excision proportion prediction models were previously built using genotyping and RNA-sequencing data from 49 tissues of European ancestry from the Genotype-Tissue Expression (GTEx) Project (v8) [41]. These models were built based on fine-mapping of cis-SNPs of each gene (or each intron) to select predicting SNPs and estimate the effect sizes of these selected SNPs by applying the multivariate adaptive shrinkage (MASH) [42] method to the marginal eQTL and sQTL effects across these 49 tissues. Specifically, building prediction models for a gene includes the following steps: (1) Only genes with cis-eQTLs with a false discovery rate of 5% in any tissues were selected. (2) Fine mapping was performed in each tissue in the corresponding cis gene region (±1 Mb of the gene) to select variants with minor allele frequency > 0.01 and posterior inclusion probabilities (PIPs) > 0.01 and genes with at least one credible set of PIP > 0.1, where the credible set PIP is sum of PIPs of variants in the set, were selected. Then in each credible set, only the variant with the highest PIP was kept. A union of selected variants across 49 tissues was obtained and linkage disequilibrium (LD) pruning was applied to the union of variants to remove redundant variants. (3) The MASH method [42] was applied to the marginal eQTL effects across the 49 tissues at the union of variants to jointly estimate effects of eQTLs. (4) The predicted expression of the gene in each tissue was calculated as the linear combination of genotypes multiplying by their estimated effect sizes of the selected variants. By a similar way as described for expression models, splicing prediction models were built for each intron in 49 tissues in GTEx (v8) samples of European ancestry. The only difference is to predict the (normalized) intron excision proportion instead of the gene expression by using cis-variants. From these prebuilt models, we specifically utilized models from 11 tissues relevant to breast cancer etiology for this study including female-specific tissues (breast, uterus, vagina, ovary), connective and fat tissues akin to those in breast (subcutaneous adipose, visceral adipose, and cultured fibroblasts), immune cell-related tissues (whole blood, EBV-transformed lymphocytes, and spleen), and liver.

**Harmonizing SNPs between expression prediction models and GWAS summary statistics.** To harmonize the variants utilized in expression prediction models with GWAS summary statistics, we utilized the ImpG-Summary [43] method to impute z-scores from the genotyping data from GTEx samples. ImpG-Summary assumes the distribution of z-scores for all SNPs at a locus approximately follows a normal distribution with 𝒁̴ 𝑁(𝟎,S) where S is the pairwise correlation matrix between all SNPs induced by linkage disequilibrium (LD); from these pairwise correlations, ImpG-Summary estimates the posterior mean of z-scores for unobserved SNPs. As input for ImpG-Summary, we utilized the correlation matrix estimated using GTEx genotyping data along with ER + and ER- summary statistics, separately.

**Joint-tissue expression- and splicing-based TWAS analyses.** In this study we utilized two joint-tissue TWAS approaches including (1) an expression-based approach and (2) a splicing-based approach. Firstly, for the expression-based TWAS approach, we performed a conventional TWAS for each gene using the S-PrediXcan software [22] separately in each of the 11 tissue types using eQTL-based prediction models. We then combined TWAS p-values for each gene across all 11 tissues using the aggregated Cauchy association test (ACAT) method [44]. The ACAT method calculates a test statistic T_ACAT_ using the following formula where p_k_ is the p-value of the k^th^ tissue type and w_k_ is the weight of that tissue type: ${\sum }_{k=1}^{11}{w}_{k}\text{t}\text{a}\text{n}\left(\right(0.5-{p}_{k})$π). In our study, we utilized equal weighting of tissues where w_k_ = 1/11. The joint p-value of this test statistic is approximated by the equation $\frac{1}{2}-(\text{a}\text{r}\text{c}\text{t}\text{a}\text{n}({\text{T}}_{\text{A}\text{C}\text{A}\text{T}})$)/π. For genes that did not have expression prediction models in all 11 tissue types, we appropriately changed the calculation of the ACAT test statistic to only include the tissue types that contained expression prediction models and modified the weighting to still be equal among the tissue type containing models for a gene. As a sensitivity analysis, we performed the ACAT analysis utilizing square root of sample size in the prediction models as the weights, and found highly similar results, with only 4 marginally significant genes additionally identified by the sample size-weighted ACAT. Thus, we kept the equal weighted results. While the (overall) gene expression measures the total expression of all isoforms of the gene, mRNA splicing (measured by intron excision ratio in a cluster) is complementary to information from total mRNA expression levels. Intron excision ratios measure the proportions of RNA-seq read counts aligned to specific excised introns in the total read counts aligned to a cluster. Hence, we additionally implemented an intron splicing-based TWAS to test association of each excised intron using S-PrediXcan with sQTL-based prediction models for introns. After performing these splicing-based TWAS analyses for individual excised introns, we then utilized ACAT to combine the p-values from all excised introns in each gene within each of our tissues to calculate a gene-based p-value in each tissue; we then performed an additional, second-step ACAT to collate gene-based p-values to obtain a joint p-value across all tissues. By implementing this splicing-based approach we may be able to identify genes that could be missed by expression-based TWAS.

**Conditioning our TWAS analyses on nearby GWAS variants.** To determine whether any genes we detected in our expression- and splicing-based TWASs were independent of previously reported GWAS signals, we performed both TWAS analyses while conditioning on genome-wide significant index SNPs (p-value < 5E-8). Specifically, we conditioned the effect sizes of SNPs (eQTL or sQTL) used in expression and splicing prediction models on nearby GWAS significant index variants within +/- 2 Mb of the transcription start or stop sites of each gene. We then utilized the conditional and joint multiple-SNP (COJO) analysis [45] method to compute the GWAS index variant-adjusted effects of each eQTL and sQTL. We then performed our expression- and splicing-based TWAS analyses with these conditioned eQTL and sQTL effect sizes similar to that described in the preceding section. Additionally, for selected genes, we queried the NHGRI-EBI GWAS Catalog [46] to identify other previously reported GWAS SNPs within the same loci, and then utilized LDpop to examine the correlation between these index SNPs and the SNPs used in our eQTL and sQTL prediction models [47].

**Conditioning GWAS variants on TWAS-identified genes.** To examine whether previous reported GWAS index variants affect breast cancer risk through genes identified in our TWAS analysis, we utilized the COJO analysis [45] method to calculate adjusted odds ratios of GWAS index variants in association with breast cancer risk, after adjusting for eQTL and sQTL used in gene or splicing predictions within the same locus. This is similar to mediation analysis, in which the adjusted odds ratios are the direct effect and the unadjusted odds ratios are the total effect. We calculated proportion mediated for each GWAS variant using the formula $\frac{direct\ effect \times (indirect\ effect-1)}{total\ effect-1}$ to indicate the extent to which the index variant’s total effect is accounted for by the nearby TWAS genes. We considered a GWAS index variant as being mediated by nearby genes if the variant had a proportion mediated > 0.5 and was no longer genome-wide significant in the COJO analysis (adjusted p-value > 5.0E-8).

**Gene-based fine-mapping of genes and intron excision events.** LD may result in false positive BC susceptibility genes being identified that are correlated with true causal genes. To identify candidate causal genes for ER + and ER- BC by accounting for LD structure, we performed gene-based statistical fine-mapping at the level of gene-trait associations using the Fine-mapping of Causal Gene Sets (FOCUS) software [48]. FOCUS controls for the genetic correlation and other pleiotropic effects induced by both LD structure and expression prediction model weights. We separately input weights for eQTLs and sQTLs used in the prediction models, an LD reference panel we computed using GTEx genotyping data, and subtype-specific summary statistics into FOCUS separately for each of the 11 tissue types. For each LD block in the genome, we identified credible sets of genes that contained the causal genes and credible sets of intron excision events using a confidence level of 90%. We additionally computed marginal PIPs in each of the 11 tissues for each gene (or each intron) within each region to be causal given the observed TWAS signals in each tissue. We classified TWAS genes that met a PIP threshold of 0.9 as candidate causal genes.

**Gene set and functional annotation enrichment analysis:** To determine gene sets with annotated biological pathways and other functional categories, we performed an enrichment analysis of protein-coding and long non-coding RNA (lncRNA) genes, separately for ER + and ER- breast cancer, using the GENE2FUNC method within the Functional Mapping and Annotation of Genome-wide association studies (FUMA) software package [49]; as the background gene set for testing enrichment of gene sets, we specified 33,527 protein-coding and lncRNA genes. We utilized a multiple testing threshold of an FDR-adjusted p-value < 0.05 for reporting significantly enriched gene sets.

## Results

**Two joint-tissue TWAS approaches: an expression-based and a splicing-based approach.** We utilized expression and splicing prediction models trained in 11 tissue types obtained from European individuals from the GTEx v8 dataset; sample sizes ranged from 129 to 670 and had weights refined using a multivariate adaptive shrinkage (MASH) method. In our TWAS analyses, we tested 19,288 genes across the 11 tissues with expression prediction models including 14,615 genes in breast tissue alone, and 14,527 genes with intron splicing prediction models including 10,928 genes in breast tissue alone.

**Figure 1:**
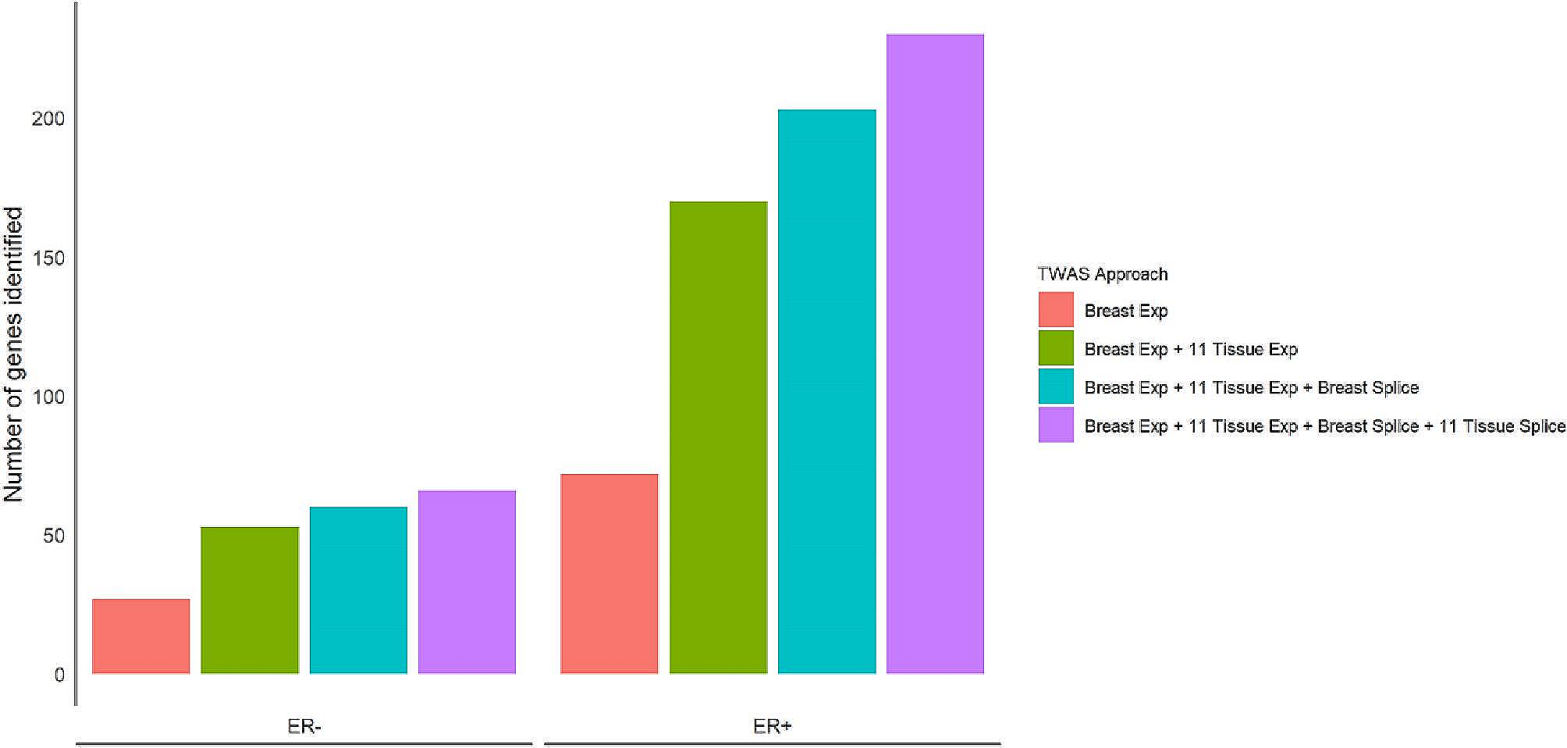
Number of genes identified cumulatively using each TWAS approach. *Abbreviations*: TWAS, transcriptome-wide association study; “Breast Exp”, genes identified using the expression-based TWAS approach in breast tissue only; “Breast Exp + 11 Tissue Exp”, genes identified using the expression-based TWAS approach in either breast tissue only or jointly across tissues; “Breast Exp + 11 Tissue Exp + Breast Splice”, genes identified using the expression-based TWAS approach in either breast tissue only or jointly across tissues and the splicing-based approach in breast tissue only; “Breast Exp + 11 Tissue Exp + Breast Splice + 11 Tissue Splice”, genes identified using the expression-based TWAS approach in either breast tissue only or jointly across tissues and the splicing-based approach in either breast tissue only or jointly across tissues

Using either the expression- or splicing-based approaches, we discovered that 230 genes were significantly associated with ER + BC at Bonferroni significance thresholds (expression-based p-value < 2.6E-6 or splicing-based p-value < 3.4E-6) (Table S2 in Additional file 1). If only considering eQTL in breast tissue, 72 (30%) genes were identified, and the number increased to 170 in multi-tissue expression TWAS, while 60 (26%) genes were identified only using the splicing-based approach (Fig. 1). Of the 230 genes associated with ER + BC, 43 genes have not been reported in any previous TWAS (**Table** 1). Among previously reported TWAS genes, 30 were identified in ER + TWAS studies and 5 in ER- TWAS studies (S1 in Additional file 1). Of the 230 ER + genes, 228 were located in 85 known GWAS loci, while one protein-coding gene (*FAM72C*) and one pseudogene (*FCGR1CP*) located at the 1q21.1, L1 locus were at least 1.4 Mb away from any previously identified GWAS risk variants (*p* < 5E-8) and were also not in LD with any risk variants (S3 in Additional file 1). Overall, among the 113 previously reported GWAS loci for ER + BC, 72 loci had a gene identified in our ER + TWAS (S3 in Additional file 1).

**Table 1:** Genes significantly associated with estrogen receptor (ER)-positive breast cancer that have not been identified in previous TWAS of breast cancer

Loci	Gene Name	Min. 11 Tissue ACAT p-value^a^	Min. Breast p-value^b^	TWAS-significant Approaches^c^	Max. PIP (eQTL)^d^	Max. PIP (sQTL)^e^
1p34.1-p33	RAD54L	5.24E-07	NA	express	0.56	NA
1p34.1-p33	UQCRH	1.48E-06	9.11E-01	express	0.06	NA
1p34.1-p33	FAAH	7.08E-09	3.39E-07	express|splice	1	0.39
1q21.1, L1	FCGR1CP	5.05E-11	NA	splice	NA	1
1q21.1, L2	ITGA10	1.46E-06	1.91E-06	splice	NA	0.36
1q21.1, L2	HJV	9.75E-07	6.46E-01	splice	NA	0.3
1q22	DPM3	2.51E-06	2.51E-06	splice	NA	0.02
1q22	ARHGEF2	4.02E-07	2.10E-04	express	0.44	NA
3p14.1	THOC7	5.21E-06	5.81E-07	splice	NA	0
3p24.1, L2	TGFBR2	3.47E-09	1.14E-02	express	1	NA
3p26.1	ITPR1	7.41E-11	3.17E-01	splice	NA	1
4q21.23	HELQ	1.16E-06	1.42E-06	splice	NA	0.93
4q34.1	RP13-577H12.2	1.97E-12	NA	splice	NA	1
5q11.1	PARP8	2.96E-06	1.08E-06	express	0	NA
5q14.3	ARRDC3	3.39E-10	9.52E-11	splice	NA	1
5q33.3	EBF1	1.61E-15	NA	splice	NA	1
6p22.1-p21.33	TRIM31	1.43E-06	7.78E-02	express	0.94	NA
6p22.1-p22.2	BTN3A1	1.93E-06	1.24E-06	splice	NA	0.87
6p22.1-p22.2	ABT1	7.41E-07	7.70E-02	splice	NA	0.74
6p22.1-p22.2	ZSCAN9	1.34E-06	9.36E-03	splice	NA	0.12
6p22.1-p22.2	PGBD1	9.86E-07	4.75E-07	splice	NA	0.48
6p22.1-p22.2	ZSCAN23	1.45E-07	8.88E-08	splice	NA	0.55
6p23	SIRT5	5.03E-06	3.31E-06	express	0.76	NA
7q21.2	ANKIB1	6.90E-07	7.75E-07	splice	NA	0.01
10p12.31-p12.2	MLLT10	2.22E-16	1.98E-02	splice	NA	0.5
10p12.31-p12.2	PIP4K2A	4.38E-07	8.95E-07	splice	NA	1
10q21.2-q21.3	EGR2	9.53E-08	9.53E-08	splice	NA	0.98
10q26.13	ENSG00000273767	1.66E-06	NA	splice	NA	0.53
11p15.5, L2	AC051649.12	2.22E-16	NA	splice	NA	0.07
11p15.5, L2	H19	2.96E-09	1.63E-09	splice	NA	0
12q24.21	RP11-116D17.3	1.06E-06	3.14E-01	express	0.95	NA
14q24.1	RAD51B	2.23E-07	8.64E-01	splice	NA	0.99
14q32.11-q32.12	CCDC88C	5.45E-10	8.72E-11	splice	NA	1
14q32.11-q32.12	PPP4R3A	4.79E-07	2.63E-02	splice	NA	0.95
15q22.33	SMAD3	2.31E-06	4.07E-02	splice	NA	0.88
15q24.1	SCAMP2	4.84E-07	3.59E-03	splice	NA	0.93
15q26.1	VPS33B	8.09E-11	6.34E-11	splice	NA	1
16q13	AMFR	2.85E-06	2.85E-06	splice	NA	0.72
17q11.2	ATAD5	5.93E-07	6.22E-01	splice	NA	0.98
18q11.2, L2	CHST9	6.74E-09	NA	splice	NA	1
19p13.13	NACC1	4.72E-07	4.72E-07	splice	NA	0.51
19q13.31	ZNF45	6.32E-12	6.69E-12	splice	NA	1
22q12.1-q12.2	CTA-292E10.6	3.01E-09	8.71E-03	splice	NA	0.9

*Abbreviations:*

TWAS, transcriptome-wide association study; ER+, estrogen receptor-positive.

^a^ The minimum joint-tissue TWAS p-value between either the expression-based or splicing-based approach.

^b^ The minimum TWAS p-value in breast tissue between either the expression-based approach or splicing-based approach.

^c^ “Express” indicates the listed gene was identified using only the expression-based TWAS approach in either only breast tissue or jointly across all tissues. “splice” indicates the listed gene was identified using only the splicing-based TWAS approach in either only breast tissue or jointly across all tissues. “express|splice” indicates the listed gene was identified in either expression-based or splicing-based approaches in either breast tissue or jointly across all tissues.

^d^ The maximum PIP of an eQTL for a given gene across 11 tissues.

^e^ The maximum PIP of an sQTL for a given gene across 11 tissues.

Furthermore, we discovered 66 genes that were significantly associated with ER- BC at Bonferroni significance thresholds (Table S2 in Additional file 1). If only considering eQTL in breast tissue, 27 (41%) genes can be identified, and the number increased to 53 in multi-tissue expression TWAS, while 13 (20%) genes were identified exclusively using the splicing-based approach (Fig. 1). Of the 66 genes associated with ER- BC, 24 genes have not been reported in any previous TWAS (Table 2), and among previously reported TWAS genes, 11 were identified in ER- TWAS studies and 6 in ER + TWAS studies (Table S1 in Additional file 1). The 66 genes we identified in the ER- TWASs were all located in 29 known BC GWAS loci. Among the 45 previously reported GWAS loci for ER- BC, 24 loci contained a gene identified in our ER- TWAS (Table S4 in Additional file 1).

**Table 2:** Genes significantly associated with estrogen receptor (ER)-negative breast cancer that have not been identified in previous TWAS of breast cancer

Loci	Gene Name	Min. 11 Tissue ACAT p-value^a^	Min. Breast p-value^b^	TWAS-significant Approaches^c^	Max. PIP (gene)^d^	Max. PIP (intron)^e^
1q32.1, L3	LGR6	8.52E-08	1.22E-08	splice	NA	1
1q32.1, L4	PIK3C2B	< 5.50E-17	1.46E-24	express	1	NA
2p23.2	WDR43	6.54E-13	7.81E-13	express|splice	1	1
2q33.1	CLK1	1.34E-08	6.97E-01	express|splice	1	0.45
2q33.1	FAM126B	6.89E-08	2.02E-04	express|splice	0.26	0.36
2q33.1	NDUFB3	2.36E-08	NA	express	0.64	NA
5q11.2, L2	PELO	2.36E-06	2.76E-05	splice	NA	0.87
5q33.3	EBF1	7.34E-07	NA	splice	NA	0.92
6p21.32	RPS18	1.49E-07	1.15E-07	express	0.98	NA
6p21.32	B3GALT4	3.38E-08	8.46E-09	express	0.9	NA
8p23.3	RPL23AP53	2.16E-06	6.86E-07	splice	NA	0.97
9p21.3	CDKN2B	1.58E-06	1.94E-01	express	0.97	NA
11q22.3	ACAT1	2.53E-06	1.99E-06	splice	NA	0.96
11q22.3	C11orf65	1.50E-06	4.92E-04	express	1	NA
16q12.2, L1	FTO	7.19E-08	NA	splice	NA	0.99
17p13.1	TP53	2.34E-06	9.92E-07	express	0.87	NA
19p13.11, L1	USE1	6.75E-09	4.03E-02	splice	NA	0
19p13.11, L1	OCEL1	3.16E-10	4.30E-10	express|splice	0.15	0
19p13.11, L1	NR2F6	1.40E-10	2.33E-11	express	0.08	NA
19p13.11, L1	USHBP1	6.11E-16	5.55E-17	splice	NA	0
19p13.11, L1	DDA1	2.96E-14	7.13E-01	express|splice	1	0
19p13.11, L1	ANO8	1.02E-13	4.86E-08	express|splice	0	1
19p13.11, L1	GTPBP3	1.52E-11	1.86E-11	splice	NA	0.33
19q13.31	ZNF45	3.53E-08	3.80E-08	splice	NA	1

*Abbreviations:*

TWAS, transcriptome-wide association study; ER-, estrogen receptor-negative.

^a^ The minimum joint-tissue TWAS p-value between either the expression-based or splicing-based approach.

^b^ The minimum TWAS p-value in breast tissue between either the expression-based approach or splicing-based approach.

^c^ “Express” indicates the listed gene was identified using only the expression-based TWAS approach in either only breast tissue or jointly across all tissues. “splice” indicates the listed gene was identified using only the splicing-based TWAS approach in either only breast tissue or jointly across all tissues. “express|splice” indicates the listed gene was identified in either expression-based or splicing-based approaches in either breast tissue or jointly across all tissues.

^d^ The maximum PIP for a given gene across 11 tissues.

^e^ The maximum PIP of introns in a given gene across 11 tissues.

**Figure 2:**
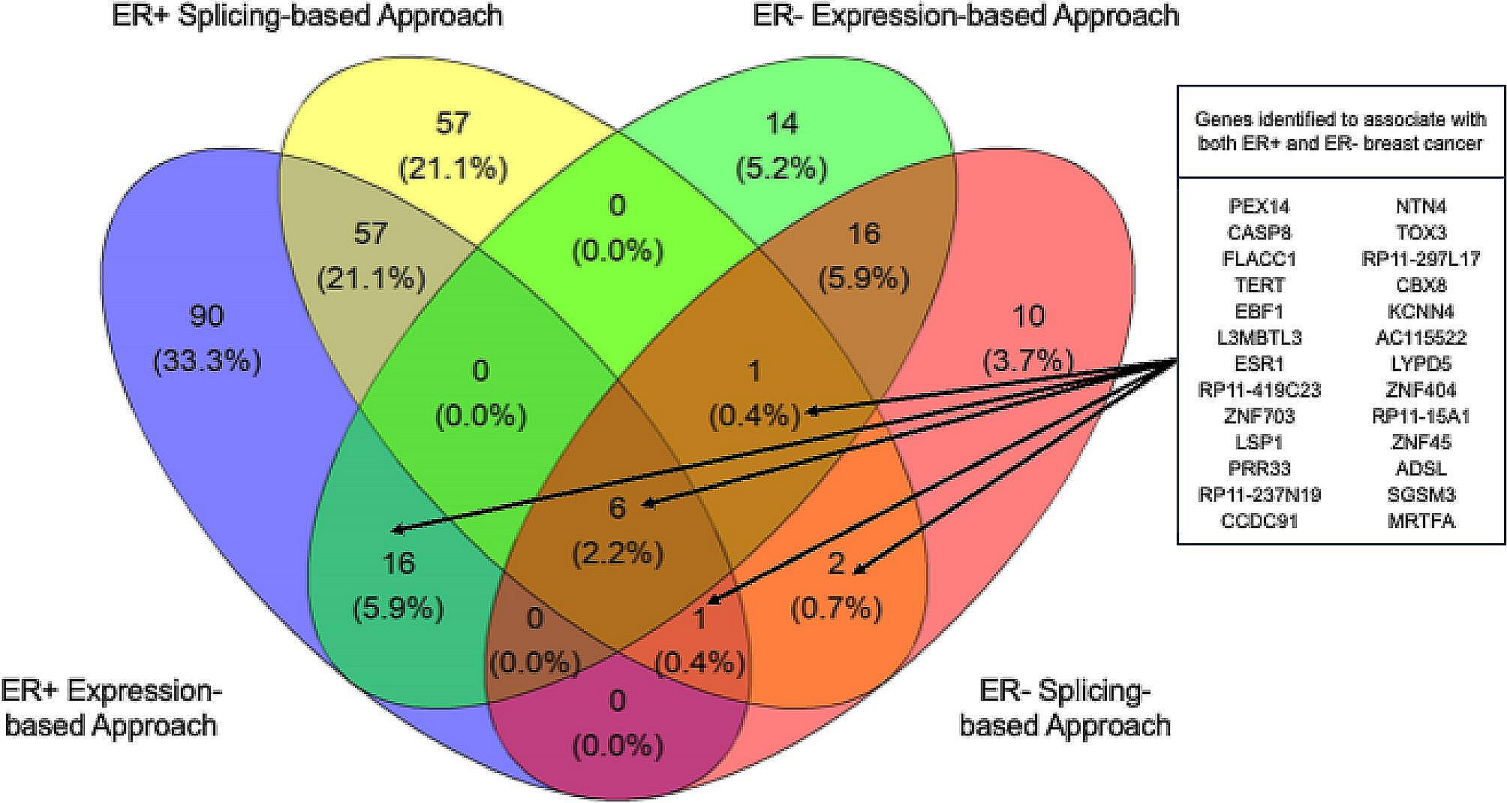
Venn diagram of TWAS genes identified for ER + and ER + breast cancer using each TWAS approach. *Abbreviations*: TWAS, transcriptome-wide association study; ER+, estrogen receptor-positive; ER-, estrogen receptor-negative

**Figure 3:**
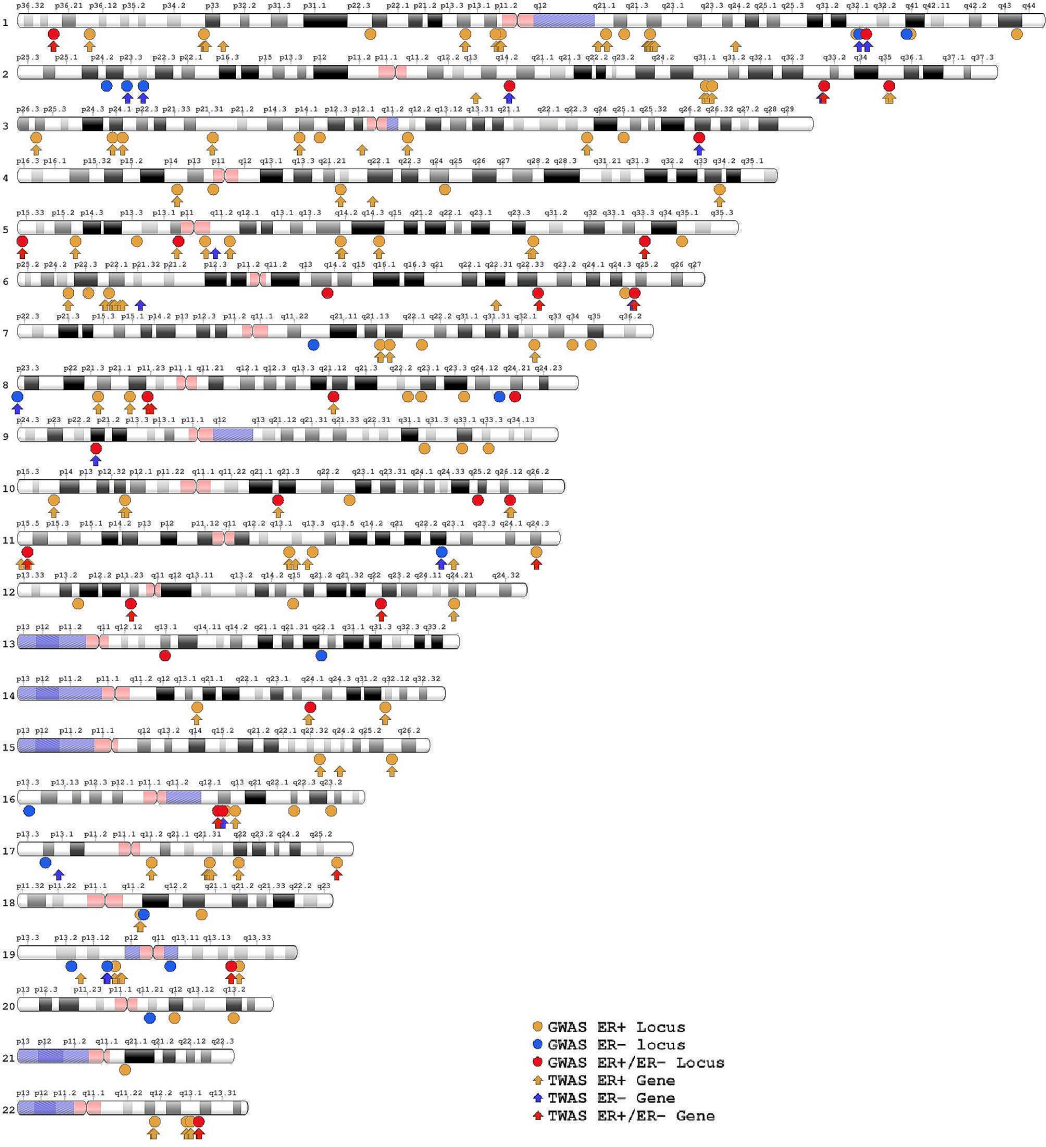
Ideogram of the 270 TWAS-identified genes for ER + and ER- breast cancer in the context of known GWAS loci of breast cancer. *Abbreviations*: TWAS, transcriptome-wide association study; GWAS, genome-wide association study; ER+, estrogen receptor-positive; ER-, estrogen receptor-negative

Overall, a vast majority of genes identified using either splicing- or expression-based approaches was unique to either the ER+ (204 genes) or ER- (40 genes) subtype with only 26 genes being associated with both ER + and ER- BC (Fig. 2). Several noteworthy genes that specifically were associated with ER + BC include *FGFR2* and *CHEK2*. Several noteworthy genes that were associated with ER- BC include *TP53* and its regulator *MDM4*. Interestingly, we discovered that *TERT* in the 5p15.33 locus was associated with both ER- and ER + BC in our TWAS, while *TERT* was previously believed to be a predominantly ER- locus in GWAS. As shown in Table S5 (in Additional file 1), SNPs annotating to *TERT* generally have larger effect sizes for ER- BC than those for ER + BC. Furthermore, we discovered that *TOX3*, which has been historically viewed as a gene implicated in ER + and overall BC risk (with an index SNPs having ORs generally above 1.2 and below 0.8 for ER + BC), was associated with both ER + and ER- BC risk in our study (Table S5 in Additional file 1). The 270 TWAS-identified genes of ER + and ER- genes were mapped across genome in the context of known GWAS loci of ER + and ER- breast cancer, showing that our TWAS study identified genes in most of the GWAS loci (Fig. 3).

**Joint TWAS analyses conditioned on BC GWAS index variants.** To test whether the association between genes identified in our TWAS analyses and breast cancer risk could be explained by nearby GWAS variants, we conditioned the effect sizes of eQTLs and sQTLs on nearby index SNPs separately for ER + and ER- BC prior to performing our TWAS analyses. After conditioning on nearby index SNPs, we discovered that 27 genes in 17 loci remained Bonferroni-significant for ER + BC and 9 genes in 6 loci remained Bonferroni-significant for ER- BC (Table 3). Among these conditionally significant genes, only two genes in two loci (*CCDC91* at 12p11.22, L1 and *TOX3* at 16q12.1-q12.2) were identified for both ER + and ER- BC. Interestingly, *CHEK2*, a gene previously reported to impact BC risk via rare and highly penetrant mutations, was identified to be conditionally significant for ER + BC (Table 3); moreover, the SNPs used in *CHEK2* expression prediction models generally had little to no correlation with other GWAS index SNPs at the same 22q12.1-q12.2 locus (Table S6 in Additional file 1).

**Table 3:** Genes significantly associated with estrogen receptor (ER)-positive and/or ER-negative breast cancer after conditioning on nearby GWAS SNPs for breast cancer risk

Loci	Gene Name	Closest GWAS Index SNP	Distance to Closest GWAS Index SNP (kb)	Min. 11 Tissue ACAT COJO p-value^a^	Min. Breast COJO p-value^b^	COJO TWAS-significant Approaches^c^
***Estrogen receptor positive***						
1q21.1, L1	FCGR1CP	rs1552172	1846.8	3.33E-11	NA	splice
1q21.1, L1	FAM72C	rs1552172	1758.3	1.25E-11	NA	express
1q21.1, L2	ITGA10	rs143384623	60.4	1.20E-06	1.58E-06	splice
2q35	DIRC3	rs6436017	102.1	2.29E-10	1.74E-01	express
3p24.1, L1	NEK10	rs4973768	5.1	1.82E-11	2.02E-12	express
5p12	MRPS30	rs930395	1.9	2.78E-16	2.78E-16	express|splice
5p12	RP11-53O19.3	rs930395	3.7	5.55E-17	9.11E-01	express
8q21.13	HNF4G	rs72658071	14.4	1.47E-10	NA	express|splice
10p12.31-p12.2	PIP4K2A	rs541079479	0	5.22E-07	6.12E-07	splice
11p15.5, L2	LSP1	rs576603	0.6	9.57E-13	2.82E-04	express
11p15.5, L2	TNNT3	rs576603	26.8	5.22E-12	5.12E-04	express|splice
11q13.3	RP11-554A11.8	rs72932540	0	3.44E-07	NA	express
12p11.22, L1	CCDC91	rs7297051	111.4	6.45E-08	5.34E-08	express|splice
14q32.11-q32.12	CCDC88C	rs150658557	4.7	8.74E-07	1.35E-07	splice
16q12.1-q12.2	TOX3	rs3803662	4.6	< 5.50E-17	2.21E-02	express
17q22	COX11	rs6504950	10.4	2.32E-08	2.97E-01	splice
18q11.2, L2	CHST9	rs232320	0	8.27E-07	NA	splice
19p13.11, L2	LRRC25	rs7258465	25.2	1.15E-09	2.19E-01	express
19p13.11, L2	SSBP4	rs7258465	0	6.34E-09	3.89E-08	splice
19p13.11, L2	ISYNA1	rs7258465	11.6	6.01E-08	3.00E-08	express
19q13.32	GIPR	rs61373376	0	1.40E-06	1.55E-02	express
22q12.1-q12.2	TTC28	rs62235681	4.9	9.72E-10	6.49E-02	express
22q12.1-q12.2	CHEK2	rs62235681	3	1.16E-12	6.57E-02	express|splice
22q12.1-q12.2	HSCB	rs17879961	16.9	2.02E-12	8.42E-08	express|splice
22q12.1-q12.2	CCDC117	rs17879961	47.6	1.46E-09	1.17E-06	express|splice
22q12.1-q12.2	XBP1	rs17879961	69.5	7.97E-09	5.37E-06	express
22q12.1-q12.2	CTA-292E10.6	rs4822992	24.2	2.91E-09	6.63E-04	splice
***Estrogen receptor negative***						
1q32.1, L4	MDM4	rs4245739	0	3.29E-09	9.86E-10	splice
2q14.2	RALB	rs4528762	27.2	4.52E-07	1.69E-07	splice
2q14.2	INHBB	rs11903787	15.5	6.09E-07	2.66E-01	express
2q33.1	FAM126B	rs13015648	54.1	5.23E-07	8.21E-03	express
2q33.1	NDUFB3	rs13015648	40.1	3.47E-08	NA	express
2q33.1	CFLAR	rs13015648	0	5.34E-06	3.40E-06	express
11q22.3	ACAT1	rs199504893	249.1	2.18E-07	4.45E-08	splice
12p11.22, L1	CCDC91	rs7297051	111.4	2.71E-07	2.32E-07	splice
16q12.1-q12.2	TOX3	rs3803662	4.6	1.38E-10	6.51E-01	express

*Abbreviations:*

TWAS, transcriptome-wide association study; ER+, estrogen receptor-positive; ER-, estrogen receptor-negative; ER+, estrogen receptor-positive; COJO, conditional & joint association analysis using GWAS summary statistics.

^a^ The minimum joint-tissue TWAS p-value conditioned on GWAS index SNPs between either the expression-based or splicing-based approach.

^b^ The minimum TWAS p-value in breast tissue conditioned on GWAS index SNPs between either the expression-based approach or splicing-based approach.

^c^ “Express” indicates the listed gene was identified using only the expression-based TWAS approach in either only breast tissue or jointly across all tissues after conditioning on nearby GWAS index SNPs. “splice” indicates the listed gene was identified using only the splicing-based TWAS approach in either only breast tissue or jointly across all tissues after conditioning on nearby GWAS index SNPs. “express|splice” indicates the listed gene was identified in either expression-based or splicing-based approaches in either breast tissue or jointly across all tissues after conditioning on nearby GWAS index SNPs.

**GWAS variants conditional on TWAS-identified genes.** To examine whether previous reported GWAS index variants affect breast cancer risk through our TWAS-identified genes, we conducted association analysis after conditional on eQTL and sQTL for GWAS variants in loci with both TWAS and GWAS signals. We found 126 ER + variants had a proportion mediated > 0.5 and were no longer genome-wide significant (Table S3 in Additional file 1), suggesting that these variants may affect ER + breast cancer risk via expression regulation of nearby TWAS genes. There were 131 ER + variants still significant in the adjusted analysis; for example, several index SNPs in the 22q12.1-q12.2 locus remained significant after adjusting for nearby genes including *CHEK2*. Of ER- GWAS index variants, effects of 40 variants were found to be mediated by nearby genes, while 38 were not (Table S4 in Additional file 1).

**Gene-based fine-mapping of associations.** To identify candidate causal genes in each tissue we performed fine-mapping of gene-trait associations separately using expression and intron splicing prediction models, LD matrices, and subtype-specific GWAS summary statistics. Of the 230 genes in 86 loci associated with ER + BC, we discovered that 133 genes in 75 loci had a PIP above 0.9 in at least one of the 11 tissues, where 104 genes were identified by gene expression-based fine-mapping, 58 by intron splicing-based fine-mapping, and 29 by both fine-mapping analysis (Table S7 in Additional file 1). Of the 66 genes in 29 loci associated with ER- BC, we found that 44 genes in 26 loci had a PIP > 0.9 in a least one tissue, where 32 genes were identified by gene expression fine mapping, 24 by intron splicing fine mapping, and 12 by both types of fine mapping (Table S8 in Additional file 1).

**Enrichment of gene sets and functional annotations.** Among the genes we discovered that were associated with ER + BC, 191 were protein-coding genes and 31 were lncRNAs. Additionally, among the genes we discovered were associated with ER- BC, 59 were protein coding genes and 6 were lncRNAs. We explored the enrichment of these lncRNA and protein-coding genes identified in the ER + and ER- TWASs using FUMA package [49]. The ER + genes were enriched for gene sets including cell cycle regulation, gland development, body fat distribution, multiple other types of cancers (e.g. lung, prostate, pancreatic, esophageal), and dysregulated immune system (Crohn’s disease, allergies) (Table S9 in Additional file 1). These results suggest that the effects of many of these ER + genes may be mediated through lifestyle factors, as well as exert pleiotropic effects on other types of cancers. In contrast, ER- genes were enriched for gene sets including numerous apoptosis pathways (caspase activation, Trail signaling, c-FLIP regulation) and other types of cancers (e.g., glioblastoma, non-glioblastoma glioma, esophageal cancer, leukemia) (Table S10 in Additional file 1). In addition, we observed that ER + and ER- genes identified in our study tended to be upregulated in female reproductive tissues such as cervix, uterus, and ovary, though this enrichment for upregulation was not statistically significant for ER- genes (Figure S1 and S2 in Additional file 2). Taken together, these enrichment results suggest that future experimental studies of how ER- genes play a role in BC etiology by impacting apoptosis are warranted, as well as additional how genes in both subtypes mechanistically exert effects in female reproductive tissues.

## Discussion

In this study of ER- and ER + breast cancer, we employed an expression-based TWAS approach utilizing models trained on overall gene expression and a splicing-based TWAS approach using models trained on intron excision events. We applied both TWAS approaches in breast tissue only, as well as jointly across 11 tissues potentially related to breast cancer development. In total, we identified 66 genes in 29 loci that were associated with ER- breast cancer and 230 genes in 86 loci that were associated with ER + breast cancer at a Bonferroni threshold of significance. In general, we observed modest consistency between our findings and genes reported in previous TWAS studies (Table S1, in Additional file 1). Among the 66 genes associated with ER- BC, 11 were reported in previous ER- TWASs and 6 in ER + TWASs. Among the 230 genes associated with ER + BC, 30 had been previously reported in TWASs of ER + BC and 5 in ER- TWASs (Table S2, in Additional file 1).

Our TWASs of ER- and ER + breast cancer identified more genes compared to previously conducted TWAS studies of breast cancer subtypes (22 ER- and 69 ER + in all previous TWAS combined). One factor leading to this higher number of identified genes was that our study had increased statistical power compared to previous studies by utilizing the latest GTEx v8 prediction models that were trained on much larger sample sizes (up to 670 participants per tissue type) [50] compared to previous TWAS studies which primarily utilized prediction models trained only on 67 GTEx breast samples [26, 30]. In addition, by utilizing predictive models from multiple tissue types, we were able to identify candidate genes that may play a role in the etiology of ER- and ER + breast cancer in tissues other than breast tissue. Given that dysregulated gene expression in tissues other than breast tissue have been associated with breast carcinogenesis through either having similar characteristics as cell types in breast – such as adipose tissue resembling adipocytes in breast – and/or having a direct impact on breast cancer etiology such immune cell-related tissues, it is no surprise that utilizing predictive expression models from multiple tissues allowed us to identify more genes than that using only breast tissue [51–53]. Furthermore, unlike previous TWAS studies which have traditionally utilized models to predict overall gene expression, our splicing-based approach additionally incorporates the predicted expression of excised introns to draw associations between gene expression and breast cancer.

Furthermore, a vast number of genes we discovered in this study were uniquely associated with either ER + or ER- BC including 40 genes that uniquely associated with ER- and 204 genes that uniquely associated with ER + BC. In particular, one gene we discovered that was only associated with ER + BC was *FGFR2*, a receptor tyrosine kinase that is involved in cell growth and proliferation [54] that has been shown to contain several variants that associated with an increased risk of both ER + and overall BC [55–57]. We identified *FGFR2* to be significant via both expression- and splicing-based TWAS approaches and tissue with the max PIP was cultured fibroblasts (PIP = 1), indicating *FGFR2* likely causally impacts breast cancer risk through its expression in fibroblasts. After conditioning on a nearby GWAS index variant rs10510097, these associations were no longer statistically significant. Moreover, one of the SNPs used to predict *FGFR2* expression across many GTEx tissues, rs1863744, was in moderate LD with rs10510097 (R^2^ = 0.40). This finding suggests that SNPs in LD with the GWAS SNP rs10510097 may partially contribute to ER + BC etiology through altered expression of *FGFR2*. Another interesting gene we identified that uniquely associated with ER- BC was *TNFSF10*. This gene was first implicated in a GWAS of ER- BC in women with African ancestry [58]; later *TNFSF10* was also implicated in a GWAS of overall breast cancer risk in women with European ancestry, but with a different risk variant [37]. It has been previously shown experimentally that editing the risk variant rs13074711, which is associated with ER- BC, leads to altered expression of *TNFSF10* and subsequent IFN-β-induced apoptosis, suggesting that the signal from *TNFSF10* at the 3q26.21 locus may play a role in BC risk through immune defense mechanisms. However, since our study identified several other tissues including visceral adipose, EBV-transformed lymphocytes, and whole blood that had PIPs of 0.949 when fine-mapping eQTLs for *TNFSF10*; we suggest further research is warranted to explore the effects of *TNFSF10* expression on ER- BC risk in these additional tissues. Overall, this study’s identification of many subtype-specific TWAS genes, in combination with the minimal overlap in genes identified for each subtype, supports the notion that the genetic etiologies of these two breast cancer subtypes are mostly distinct.

In our study, we discovered several genes including *CHEK2* and *TP53* that have historically been thought to contribute to breast cancer risk through rare, moderately or highly penetrant mutations and have thus far not been identified in other TWAS. CHEK2 is an enzyme involved in apoptosis in response to double stranded DNA damage [59] that was associated only with ER + BC in both our expression- and splicing-based TWAS. Protein-truncating or rare missense variants in coding regions of *CHEK2*, especially 1100delC (rs555607708), have been previously reported to have moderate penetrance for overall breast cancer risk and ER + breast cancer in familial studies [60] and population based studies [61–63]. We noted that the 12 SNPs used to predict *CHEK2* expression across the 11 tissues had little to no correlation with rs555607708 (r^2^ < 0.009), as well as with the other 18 reported GWAS index SNPs at the 22q12.1-q12.2 locus (Table S6 in Additional file 1) in the NHGRI-EBI GWAS Catalog [46]. Also these GWAS index SNPs remained statistically significant after adjusting for eQTL/sQTL of nearby genes including *CHEK2* (Table S3 in Additional file 1*).* Together, the discovery of *CHEK2* in our ER + TWAS, alongside the lack of overlap and correlation between SNPs used in expression prediction models and GWAS index variants, suggest that common variants which modulate *CHEK2* expression likely play a role in breast cancer risk, and these common variants are etiologically distinct from those identified in previous GWAS studies. These findings strongly support the notion that there may be a previously unidentified, polygenic basis by which *CHEK2* expression contributes to the risk of developing ER + breast cancer.

While rare protein-truncating and missense mutations in *TP53* have been shown to exhibit moderate to high penetrance for both ER + and ER- breast cancer, they have only been implicated in prior GWAS but not TWAS studies of breast cancer [14, 38, 61, 63]. Even though *TP53* has been recognized as the most common somatically mutated gene in ER- breast tumors [64], our expression-based TWASs of ER- breast cancer for the joint analysis of 11 tissues as well as breast tissue alone were the first TWAS to identify *TP53.* In addition, the PIP of 0.87 in breast and 0.87 in cultured fibroblasts for *TP53* were relatively high, which are corroborated by numerous experimental studies showing that the expression of *TP53* in these two tissues impacts breast cancer etiology [65, 66]. Moreover, the model SNP rs78378222 which was used to predict overall *TP53* expression, as well as of excised introns, across breast and several other tissues has been previously reported to associate with breast cancer risk [67]. These findings indicate that while missense and protein-truncating variants in *TP53* have historically been observed to impact breast cancer risk, common variants which modulate *TP53* expression can additionally contribute to the development of ER- breast cancer.

Our study identified 26 genes that were associated with both ER- and ER + subtypes, including *TERT* and *TOX3* which have not been reported in prior TWAS studies; both genes were identified using our expression-based approaches. Though *TERT* has been primarily regarded as an ER- gene since several GWAS variants in *TERT* have been associated with ER- BC [16, 68], our study shown that the predicted expression of *TERT* was also significantly associated with ER + breast cancer risk. As these TWAS signals for both subtypes were no longer significant in our COJO analysis, the observed signals may be explained by nearby GWAS index SNPs. On the other hand, *TOX3* has generally been regarded as a gene associated with ER + breast cancer since higher mRNA and protein expression of *TOX3* have been observed in ER + BC cell lines compared to ER- cell lines, as well as larger SNP effect sizes for the risk of developing ER + compared to ER- BC [69–71]. We additionally discovered *TOX3* expression significantly associated with ER- BC in our study. For both ER+/- BC, *TOX3* had a PIP of 1 in breast mammary tissue, supporting the notion that *TOX3* causally impacts breast cancer etiology.

While our study was robust in replicating many previously identified susceptibility loci, our approach had several limitations. First, even though our splicing-based TWAS allowed us to combine the p-values for the associations between different intron excision events, this method does not explicitly account for the direction of association between each intron excision event and BC risk. Future work into refining the splicing-based approach to account for directionality prior to combining p-values from alternatively spliced transcripts may help increase the power to detect breast cancer associated genes. Additionally, given that our study incorporated a meta-analysis and predictive expression models that both utilized primarily individuals with European ancestry, our findings may not be portable to individuals with non-European ancestral backgrounds. It has been shown that molecular subtypes, gene expression, and germline/somatic variants in breast cancer patients differ significantly between racial groups [72], and it is imperative that future TWAS studies include individuals with diverse ancestral backgrounds.

## Conclusions

In summary, our study identified many genes that are associated with ER + and ER- BC that have not been previously identified in TWAS by utilizing two joint-tissue TWAS approaches. More importantly, most of the genes for ER + and ER- breast cancer are distinct. We also discovered one novel loci for ER + BC. Interestingly, though several breast cancer susceptibility genes including *TP53* and *CHEK2* have been historically thought to play a role in breast cancer through rare, highly penetrant mutations in coding regions, our study provides evidence that common variants in these genes which modulate expression also impact breast cancer etiology. Taken together, utilizing a comprehensive combination of expression- and splicing-based methods can help improve our understanding of breast cancer genetics. Functional characterization of these candidate genes, in particular genes significant in the fine-mapping analysis, could shed some light on the etiology of ER + and ER- breast cancer as well as provide targets for treatment of breast cancer. eQTLs and sQTLs that are associated with expression of these candidate genes may be used in building polygenic risk prediction models to assess ER + and ER- breast cancer risk separately and to guide risk-adaptive breast cancer screening.

### Electronic supplementary material

Below is the link to the electronic supplementary material.

Additional file 1: Table S1. List of genes identified in previous transcriptome-wide association studies of estrogen receptor-(ER) positive or negative breast cancer. Table S2. All genes identified in this joint TWAS study for ER + and ER- breast cancer. Table S3. List of known index SNPs of ER + breast cancer GWAS and ER + genes identified in our study, by genomic locus. Table S4. List of known index SNPs of ER- breast cancer GWAS and ER- genes identified in our study. Table S5. Nearby GWAS index SNPs of TERT and TOX3. Table S6. Nearby GWAS index SNPs of CHEK2 and TP53 and their correlations with SNPs included in expression prediction models. Table S7. Candidate causal genes for ER + breast cancer identified by FOCUS. Table S8. Candidate causal genes for ER- breast cancer identified by FOCUS. Table S9. Significant gene sets in the enrichment analysis of ER + genes using FUMA. Table S10. Significant gene sets in the enrichment analysis of ER- genes using FUMA.

Additional file 2: Figure S1. Enrichment of ER + breast cancer genes for GTEx tissues. Figure S2. Enrichment of ER- breast cancer genes for GTEx tissues.

## Data Availability

In this study, we used only existing datasets that are publicly available (see web resources). The code pipeline for our TWAS analyses are available at https://github.com/shugamoe/acat_brca. For specific method code, we made minor modifications to S-PrediXcan to combine results with ACAT (https://github.com/shugamoe/MetaXcan/tree/catch_up). We also made minor modifications to FOCUS to accommodate PrediXcan GTEx v.8 MASHR models (https://github.com/shugamoe/focus). **Web resources**. BCAC summary statistics, https://bcac.ccge.medschl.cam.ac.uk/bcacdata/oncoarray/oncoarray-and-combined-summary-result. COJO (GCTA), https://yanglab.westlake.edu.cn/software/gcta/. FOCUS, https://github.com/bogdanlab/focus. FUMA, http://fuma.ctglab.nl. GTEx Portal, https://gtexportal.org/home/. PrediXcan GTEx v.8 MASHR models, https://predictdb.org/. S-PrediXcan, https://github.com/hakyimlab/MetaXcan and https://github.com/hakyimlab/summary-gwas-imputation.

## Declarations

### List of abbreviations

Estrogen receptor (ER).

Transcriptome-wide association studies (TWAS).

Breast cancer (BC).

Estrogen receptor-positive (ER+).

Estrogen receptor-negative (ER-).

Breast Cancer Association Consortium (BCAC).

Consortium of Investigators of Modifiers of BRCA1 and BRCA2 (CIMBA).

Genome-wide association studies (GWAS).

Expression quantitative trait loci (eQTLs).

Single nucleotide polymorphism (SNP).

Splicing quantitative trait loci (sQTLs).

Genotype-Tissue Expression Project (GTEx).

Collaborative Oncological Gene-Environment Study (iCOGS).

Aggregated Cauchy association test (ACAT).

Conditional and joint multiple-SNP (COJO) analysis.

Fine-mapping of Causal Gene Sets (FOCUS).

Posterior inclusion probabilities (PIPs).

Long non-coding RNA (lncRNA).

Functional Mapping and Annotation of Genome-wide association studies (FUMA).

Multivariate adaptive shrinkage (MASH).

Linkage disequilibrium (LD).
