# Supplementary material for: Expression- and splicing-based multi-tissue transcriptome-wide association studies identified multiple genes for breast cancer by estrogen-receptor status
Source: Breast Cancer Res. 2024 Mar 21;26:51. doi: 10.1186/s13058-024-01809-6 (PMC10958972; doi:10.1186/s13058-024-01809-6)
Supplement: Supplementary file 2 — Additional file 2: Figure S1. Enrichment of ER + breast cancer genes for GTEx tissues. Figure S2. Enrichment of ER- breast cancer genes for GTEx tissues. [file 13058_2024_1809_MOESM2_ESM.pptx]

## Slide 1
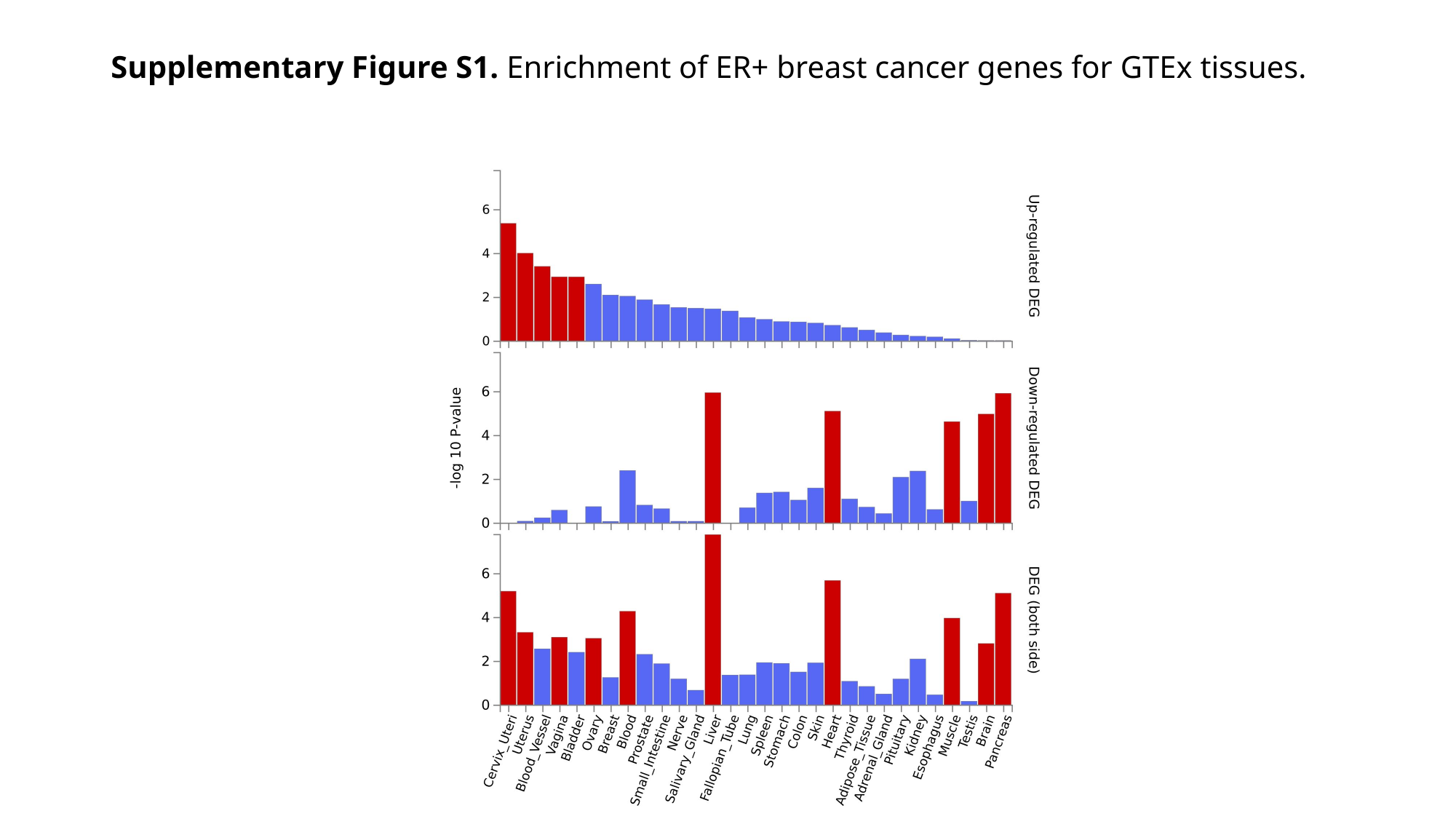

# Supplementary Figure S1. Enrichment of ER+ breast cancer genes for GTEx tissues.

## Slide 2
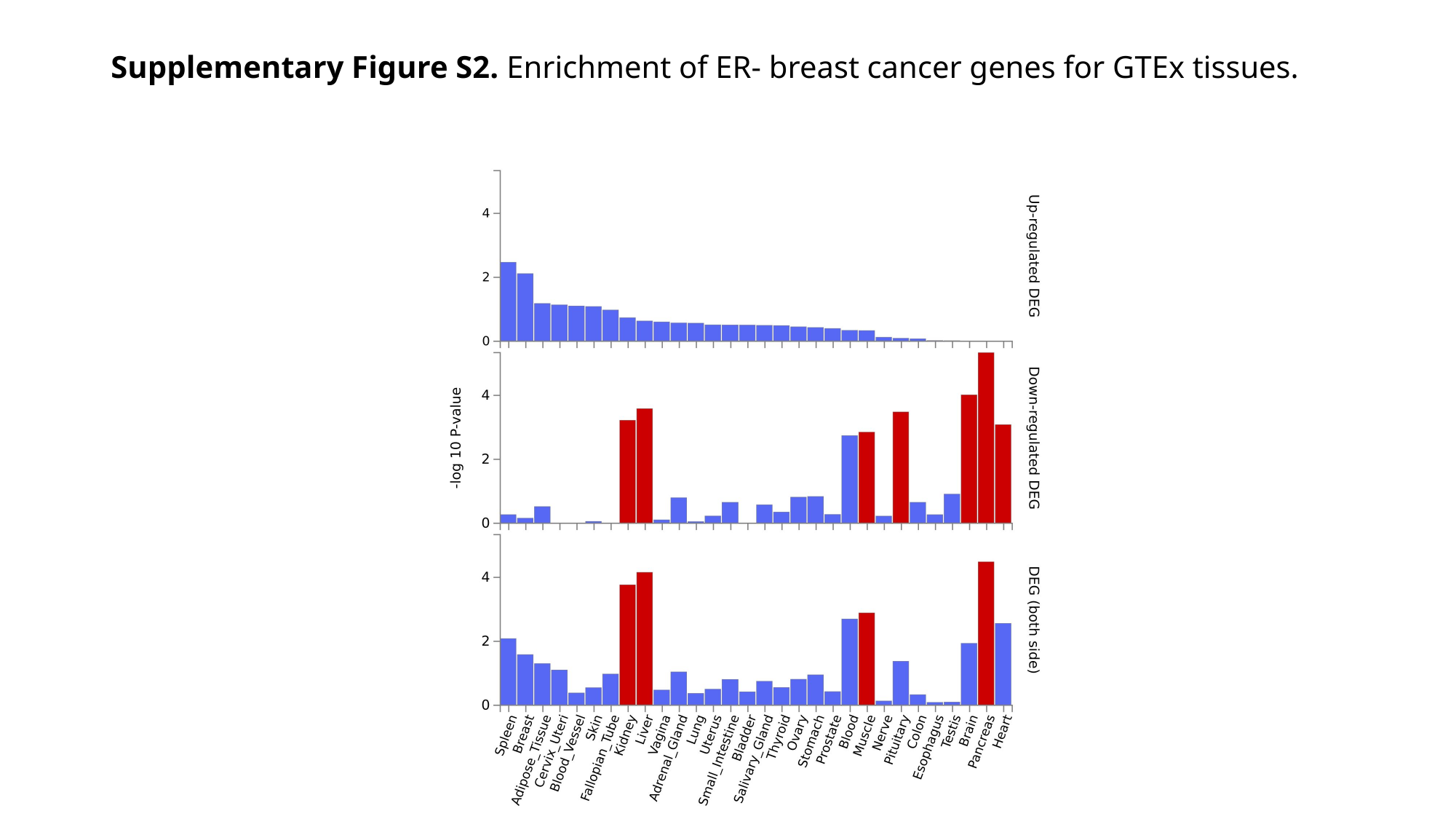

# Supplementary Figure S2. Enrichment of ER- breast cancer genes for GTEx tissues.
